# FAM46C inhibits cell proliferation and cell cycle progression and promotes apoptosis through PTEN/AKT signaling pathway and is associated with chemosensitivity in prostate cancer

**DOI:** 10.18632/aging.103030

**Published:** 2020-04-13

**Authors:** Libin Ma, Huadong He, Kang Jiang, Peiwu Jiang, Han He, Shengjia Feng, Kean Chen, Jia Shao, Gang Deng

**Affiliations:** 1 Department of Nephrology, Sir Run Run Shaw Hospital, Zhejiang University School of Medicine, Hangzhou 310016, Zhejiang, China; 2 Department of Urology, Affiliated Hangzhou First People's Hospital, Zhejiang University School of Medicine, Hangzhou 310006, Zhejiang, China; 3 Surgical Department I, Hangzhou Hospital of Traditional Chinese Medicine, Hangzhou 310007, Zhejiang, China; 4 Clinical Medical College of Zhejiang Chinese Medical University, Hangzhou 310006, Zhejiang, China; 5 Department of Urology, The Second Hospital of Jiaxing, Jiaxing 314001, Zhejiang, China

**Keywords:** prostate cancer, tumorigenesis, ubiquitination, FAM46C, PTEN

## Abstract

Family with sequence similarity 46 member C (FAM46C) is a non-canonical poly(A) polymerase that is associated with tumorigenesis. However, its role in prostate cancer development is not fully understood. Herein, we determined expression pattern of FAM46C in prostate cancer and further identified its effect on the tumorigenesis and chemosensitivity. FAM46C expression was decreased in prostate cancer tissues and cell lines compared with corresponding controls. FAM46C expression was significantly associated with the Gleason score, tumor size and overall survival. FAM46C knockdown in 22RV1 and DU145 cells significantly inhibited apoptosis and promoted cell proliferation and cell cycle progression as well as activation of AKT. FAM46C overexpression had an inverse effect in DU145 cells and inhibited tumor growth *in vivo*. FAM46C inhibited cell proliferation and cell cycle progression and induced apoptosis via the PTEN/AKT signaling pathway. FAM46C promoted PTEN expression through inhibiting PTEN ubiquitination. The prostate cancer cells and patient-derived xenograft (PDX) mice with high-FAM46C-expressing demonstrated an enhanced chemosensitivity to docetaxel. These findings suggest that FAM46C control cell proliferation, cell cycle and apoptosis through PTEN/AKT signaling pathway and is associated with chemosensitivity of prostate cancer. Modulation of their levels may offer a new approach for improving anti-tumor efficacy for chemotherapeutic agents in prostate cancer.

## INTRODUCTION

Prostate cancer is the fifth highest incidence of malignant tumor worldwide and accounts for the second highest mortality in male malignant tumors, surpassed only by lung cancer [1]. In urogenital system tumors, it exceeded bladder cancer and became the most common malignancy in men. Although the mortality and incidence of prostate cancer is relatively low in the world, they are also increasing due to the change of lifestyle, the aging of population, and the limited level of medical diagnosis [2]. The overall 5-year relative survival rate for prostate cancer is 96.5% [3]. Chemotherapy is currently the most effective method for the treatment of advanced and metastatic prostate cancer. Docetaxel has remained as a first-line cytotoxic treatment for prostate cancer for more than ten years [4]. However, only about 50% of patients respond to docetaxel, and responders eventually develop resistance [5]. Therefore, alternative therapies targeting cancer cells are an appropriate option.

Family with sequence similarity 46, member C (FAM46C) belongs to a family of four genes (FAM46A, FAM46B, FAM46C and FAM46D) and is a novel eukaryotic non-canonical poly(A) polymerase which enhances mRNA stability and gene expression. Mainly targets mRNAs encoding endoplasmic reticulum-targeted protein and may be involved in induction of cell death. FAM46A expression was increased in glioma patients and cisplatin-resistant gastric cancer cell lines and associated with pathological grade, overall survival and progression-free survival of glioma patients [6, 7]. FAM46B expression was decreased in prostate cancer patients and inhibited cell proliferation and cell cycle progression through ubiquitination of β-catenin [8]. Deletions of FAM46C have been found in a few cancers, including multiple myeloma [9], gastric cancer [10] and myeloma [11], and to be associated with a shorter overall survival. FAM46C was downregulated in hepatocellular carcinoma (HCC), induced cell apoptosis and cell cycle arrest at G2/M phase and inhibited HCC proliferation through regulating Ras/MEK/ERK pathway [12], and restrained HCC metastasis through regulating TGF-β/Smad and EMT process [13]. The deletion of FAM46C was identified as an independent risk factor for hepatic recurrence in patients with gastric cancer after curative gastrectomy [10]. FAM46C was downregulated in pancreatic ductal adenocarcinoma patients with skin rash and involved in EGFR and IFN signaling processes [14], contributing to the onset of autoimmune diseases [15]. Due to FAM46D’s restricted expression pattern and immunogenicity it represents a novel target for cancer immunotherapy [16]. However, the pathological function of these FAM46 family proteins in prostate cancer is not fully understood.

PTEN is a tumor suppressor with phosphatase activity and decrease or loss of PTEN expression, due to the methylation, mutation or deletion, may closely relate to the occurrence and development of multiple cancers [17, 18]. The clinical study found that the expression of PTEN protein in prostate cancer tissues was significantly lower than that in benign prostatic hyperplasia and negatively correlated to clinical Gleason score, pathological grade, and stage of prostate cancer [19, 20], suggesting that PTEN protein decreases with the increase of the malignancy of prostate cancer. PI3K/AKT signaling activation can inhibit chemosensitivity and cell apoptosis and accelerate cell cycle progression, angiogenesis and cell invasion, while excessive PTEN expression can inhibit the activation of PI3K/AKT that inhibits P27 and Caspase-9 expression and promotes the translation from G1 to S phase [21–24]. Prostate cancer LNCaP cells exhibited PTEN inactivation, leading to constitutive activation of the AKT pathway [25]. PTEN induction in LNCaP cells significantly induced cell cycle G1 phase arrest and inhibited cell proliferation through AKT signaling pathway [26]. These studies suggest that PTEN/AKT signaling pathway is of great importance in the process of prostate cancer. However, the involvement of PTEN/AKT signaling pathway in the function of FAM46C regulating prostate cancer is not fully understood.

In this study, we have evaluated the biological functions of FAM46C in the apoptosis, cell cycle and proliferation of prostate cancer cells and the understanding molecular mechanism involved. Downregulation of FAM46C and PTEN was found in human prostate cancer tissues. FAM46C promoted cell apoptosis and inhibited cell cycle and cell proliferation of prostate cancer through PTEN/AKT signaling pathway. FAM46C promoted PTEN expression through inhibiting PTEN ubiquitination. Higher FAM46C expression enhanced the chemosensitivity to docetaxel in prostate cancer cells and PDX mice.

## RESULTS

### FAM46C expression was downregulated and associated with Gleason score in prostate cancer tissues

**Figure 1:**
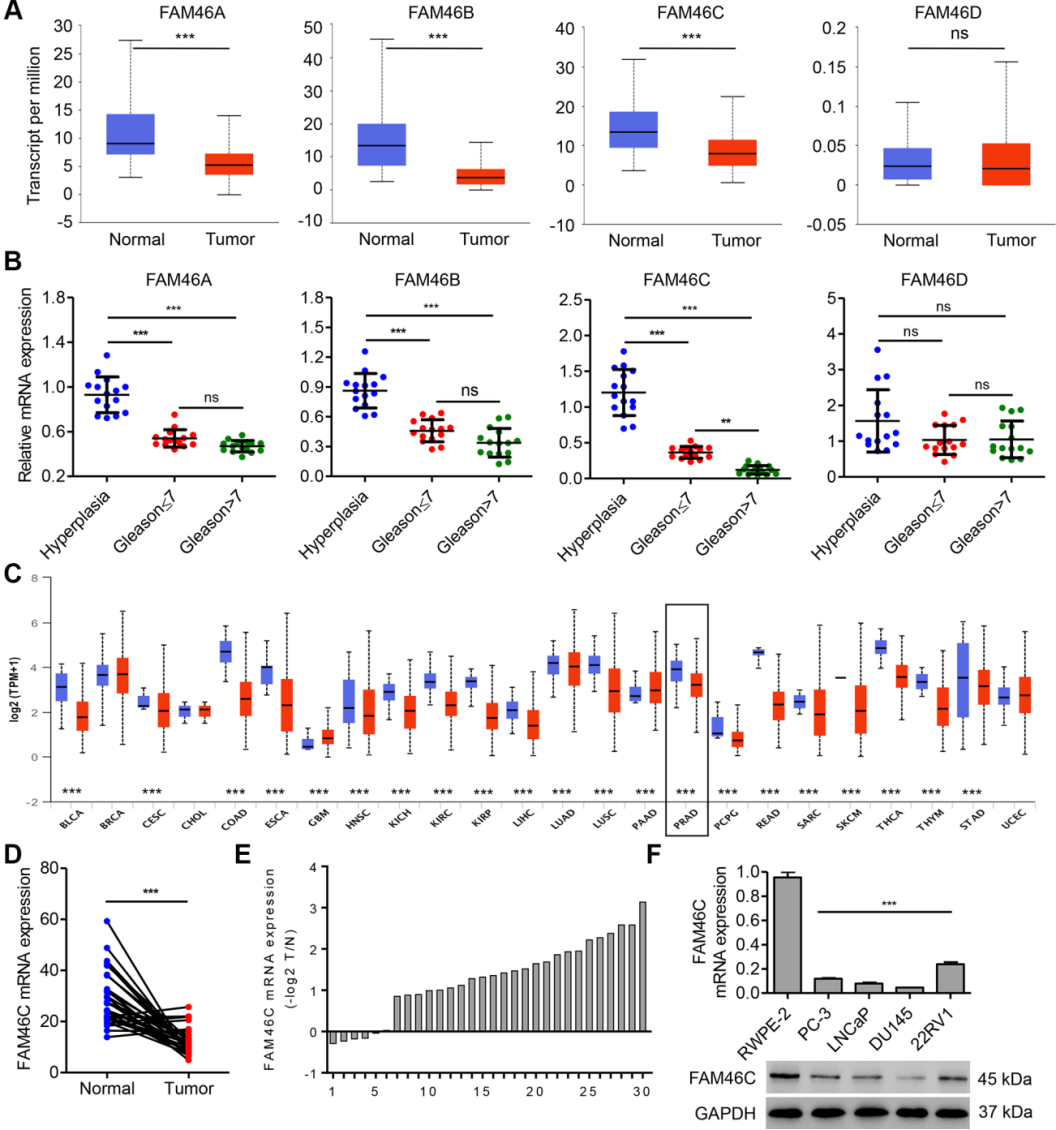
**FAM46C expression was decreased in prostate cancer tissues.** FAM46A, FAM46B FAM46C and FAM46D mRNA expression levels in prostate cancer tissues (n=52) and noncancerous prostate tissues (n=497) from TCGA database (**A**), and that in benign prostatic hyperplasia tissues and prostate cancer tissues with different Gleason scores (n=15 per group) from hospital cohort were measured by qPCR (**B**). (**C**) FAM46C mRNA expression levels in several cancer types compared with the corresponding normal controls, including bladder carcinoma (BLCA), breast carcinoma (BRCA), cervical cancer (CESC), cholangiocarcinoma (CHOL), colon and rectal adenocarcinoma (COAD), esophageal cancer (ESCA), head and neck squamous cell carcinoma (HNSC), kidney chromophobe (KICH), kidney renal clear cell carcinoma (KIRC), kidney papillary cell carcinoma (KIRP), liver hepatocellular carcinoma (LIHC), lung adenocarcinoma (LUAD), lung squamous cell carcinoma (LUSC), pheochromocytoma and paraganglioma (PCPG), prostate adenocarcinoma (PRAD), rectum adenocarcinoma (READ), sarcomas (SARC), skin cutaneous melanoma (SKCM), thyroid carcinoma (THCA), thymoma (THYM), stomach adenocarcinoma (STAD) and uterine cervical and endometrial carcinoma (UCEC), from TCGA database. Blue, normal samples. Red, tumor samples. (**D**) FAM46C mRNA expression levels in prostate cancer tissues and corresponding adjacent noncancerous prostate tissues (n=30) from hospital cohort were measured by qPCR. (**E**) Relative mRNA expression of FAM46C in 30 cases of prostate cancer patients (-log_2_T/N). (**F**) FAM46C expression levels in human prostate cancer cell lines (LNCaP, PC-3, 22RV1 and DU145) and human prostate epithelial cell line RWPE-2 were measured by qPCR and western blot. ***P < 0.001.

Bioinformatics analysis based on the TCGA database showed that the transcript level of FAM46A, FAM46B and FAM46C, but not FAM46D, was decreased in prostate cancer tissues compared with noncancerous prostate tissues (Figure 1A). Moreover, the mRNA expression of FAM46C, but not FAM46A, FAM46B and FAM46D, in high Gleason score samples was found to be lower than that in low Gleason score samples in hospital cohort (Figure 1B). These data suggest that FAM46C may involve in the prostate cancer progression, and we therefore focused on FAM46C. Bioinformatics analysis based on the expression of FAM46C across TCGA cancers (with tumor and normal samples) further showed that FAM46C expression was prevalently down-regulated in several cancer types compared with the corresponding normal controls (Figure 1C), including bladder carcinoma (BLCA), cervical cancer (CESC), colon and rectal adenocarcinoma (COAD), esophageal cancer (ESCA), head and neck squamous cell carcinoma (HNSC), kidney chromophobe (KICH), kidney renal clear cell carcinoma (KIRC), kidney papillary cell carcinoma (KIRP), liver hepatocellular carcinoma (LIHC), lung adenocarcinoma (LUAD), lung squamous cell carcinoma (LUSC), pheochromocytoma and paraganglioma (PCPG), prostate adenocarcinoma (PRAD), rectum adenocarcinoma (READ), sarcomas (SARC), skin cutaneous melanoma (SKCM), thyroid carcinoma (THCA), thymoma (THYM), and stomach adenocarcinoma (STAD). Indeed, the mRNA levels of FAM46C were significantly lower in the prostate cancer tissues compared with the noncancerous prostate tissues in hospital cohort (Figure 1D). The FAM46C mRNA levels were also shown in 30 cases of prostate cancer patients (Figure 1E). Furthermore, FAM46C expression in prostate cancer cell lines 22RV1, PC-3, LNCaP, and DU145 in addition to the human prostate epithelial cell line RWPE-2 were also measured. By comparing the expression of FAM46C in cells, we revealed that, prostate cancer cell lines demonstrated a lower FAM46C expression compared with human prostate epithelial cell line RWPE-2, and 22RV1 cells had the higher expression of FAM46C while DU145 cells had the lower expression of FAM46C (Figure 1F). So we selected these two cell lines for subsequent experiments.

### FAM46C expression was correlated with clinical factors in prostate cancer

**Figure 2:**
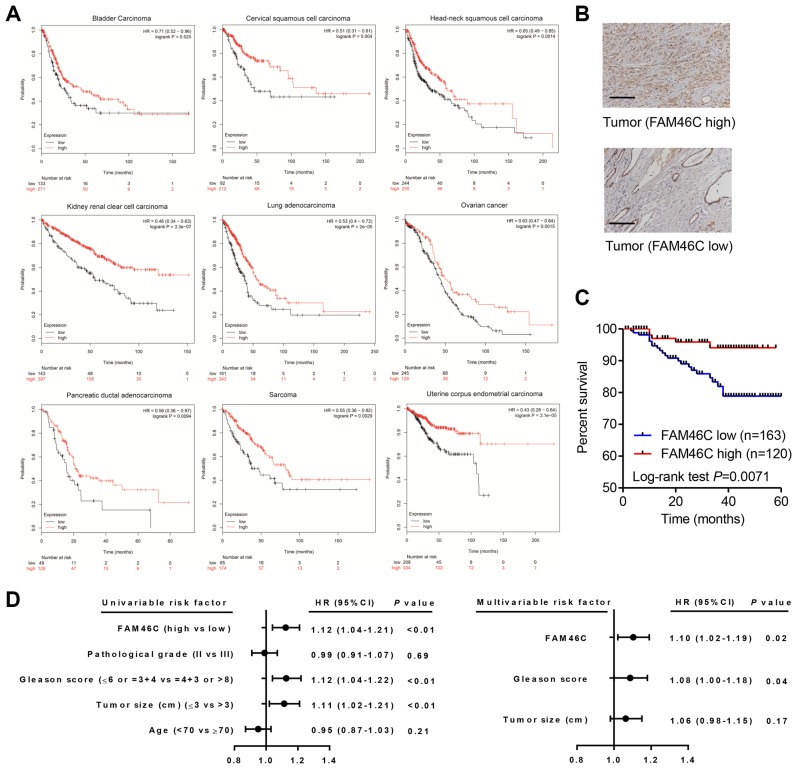
**FAM46C was a prognosis factor in prostate cancer patients.** (**A**) FAM46C expression was associated with survival outcome in several cancer types from Kaplan Meier-plotter database. (**B**) FAM46C protein expression levels in prostate cancer tissues from hospital cohort were measured by immunohistochemistry. Scale bars: 100 μm. (**C**) Kaplan-Meier curves indicated that overall survival of prostate cancer patients from hospital cohort was associated with FAM46C expression level. (**D**) Univariate and multivariate analysis of overall survival in prostate cancer patients.

Bioinformatics analysis based on the Kaplan Meier-plotter database showed that high FAM46C expression was significantly related to an increased risk for favorite clinical outcome in patients with BLCA, cervical squamous cell carcinoma (CSCC), HNSC, KIRC, LUAD, ovarian cancer (OC), pancreatic ductal adenocarcinoma (PDAD), SARC, or uterine corpus endometrial carcinoma (UCEC) (Figure 2A), suggesting that FAM46C may commonly act as a prognosis factor in cancers; however, its role in prostate cancer remains unclear. To analyze the function of FAM46C in prostate cancer, we determined FAM46C protein expression in 283 cases of prostate cancer (Figure 2B). Immunohistochemistry analysis found that 42.4% (120/283) cases demonstrated higher FAM46C expression, while 57.6% (163/283) cases demonstrated lower FAM46C expression. Patients with prostate cancer in the FAM46C high expression group were also proved to have better overall survival compared with those in the FAM46C low expression group (Figure 2C). Moreover, it demonstrated that the expression of FAM46C was correlated with the Gleason score and tumor size, but no significant difference could be found regarding the age and pathological grade of patients between FAM46C low and high expression group (Table 1). In terms of overall survival, univariate along with multivariate analysis revealed that FAM46C expression, Gleason score and tumor size were prognostic factors, and FAM46C expression as well as Gleason score was an independent prognostic factor (Figure 2D).

**Table 1:** Correlation of the expression of FAM46C with clinicopathological parameters in patients with prostate cancer.

**Characteristics**	**FAM46C expression**		***P* -value**
	**High (n=120)**	**Low (n=163)**	
Age (years)			0.8298
<70	50	70	
≥70	70	93	
Gleason score			0.0046
≤6 or =3+4	72	70	
=4+3 or ≥8	48	93	
Pathological grade			0.5706
II	70	92	
III	50	71	
Tumor size			0.0151
≤3 cm	72	74	
>3 cm	48	89	

Differences between groups were done by the Chi-square test.

### FAM46C knockdown promoted prostate cancer cell growth

**Figure 3:**
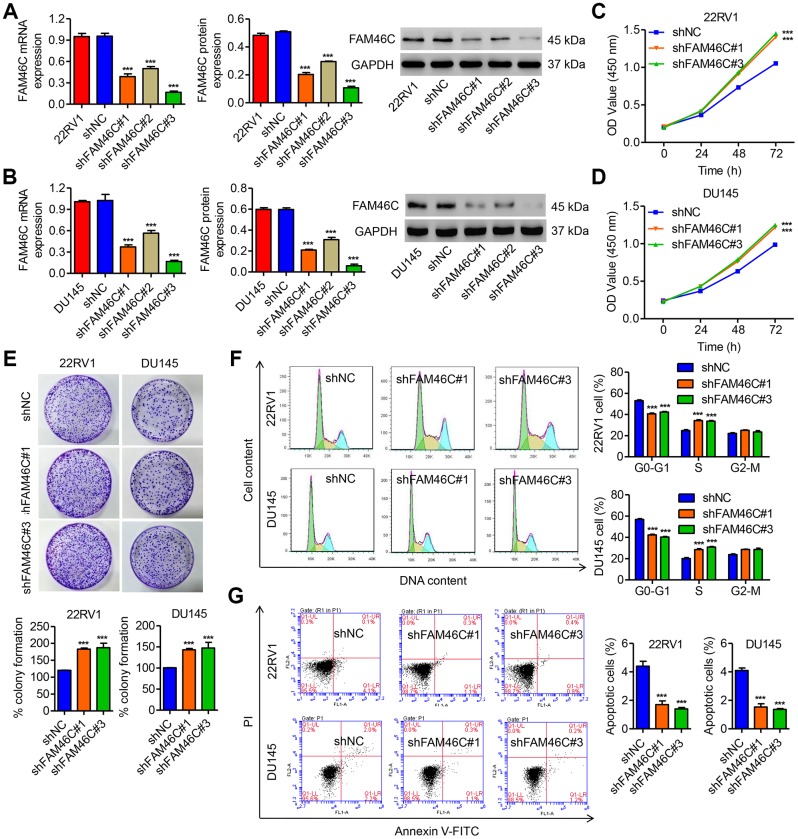
**FAM46C knockdown promoted cell growth of 22RV1 and DU145 cells.** (**A**, **B**) The efficiency of three pLKO.1-shRNAs in silencing endogenous FAM46C in 22RV1 and DU145 cells was measured by qPCR and western blot. After 22RV1 and DU145 cells were transduced with pLKO.1-shFAM46C#1 and pLKO.1-shFAM46C#3, the cell proliferation (**C**–**E**), cell cycle (**F**) and apoptosis (**G**) were measured by CCK-8, colony formation and flow cytometry, respectively. ***P<0.001 compared with pLKO.1-shNC.

To assess the role of FAM46C in prostate cancer development, we then transduced pLKO.1-FAM46C shRNAs or pLKO.1-scramble control shRNA (shNC) vector into the 22RV1 and DU145 cells (Figure 3A and 3B). pLKO.1-shRNA#1 and pLKO.1-shRNA#3 transduction resulted in lower FAM46C expression compared to pLKO.1-shRNA#2 and were therefore chosen for further experiments. Our results observed that pLKO.1-shFAM46C#1 and pLKO.1-shFAM46C#3 markedly promoted the cell proliferation of 22RV1 cells by 12.6% and 15.3% at 24 h, by 24.2% and 27.5% at 48 h, and by 33.1% and 37.8% at 72 h, respectively, compared with pLKO.1-shNC (Figure 3B). A colony-formation assay showed that pLKO.1-shFAM46C#1 and pLKO.1-shFAM46C#3 significantly promoted the colony forming growth of 22RV1 cells by 62.4% and 66.4%, respectively, compared with pLKO.1-shNC (Figure 3C). Moreover, pLKO.1-shFAM46C#1 and pLKO.1-shFAM46C#3 significantly induced the decrease of the cell number in G0-G1 phase by 23.4% and 20.3% and increase of the cell number in S phase by 37.9% and 35.8%, respectively, compared with pLKO.1-shNC (Figure 3D). pLKO.1-shFAM46C#1 and pLKO.1-shFAM46C#3 also inhibited 22RV1 cell apoptosis by 61.4% and 68.2%, respectively, compared with pLKO.1-shNC (Figure 3E). The similar results were also observed in DU145 cells with pLKO.1-shFAM46C#1 or pLKO.1-shFAM46C#3 transduction (Figure 3D–3G).

### FAM46C overexpression inhibited prostate cancer cell growth *in vitro* and *in vivo*

**Figure 4:**
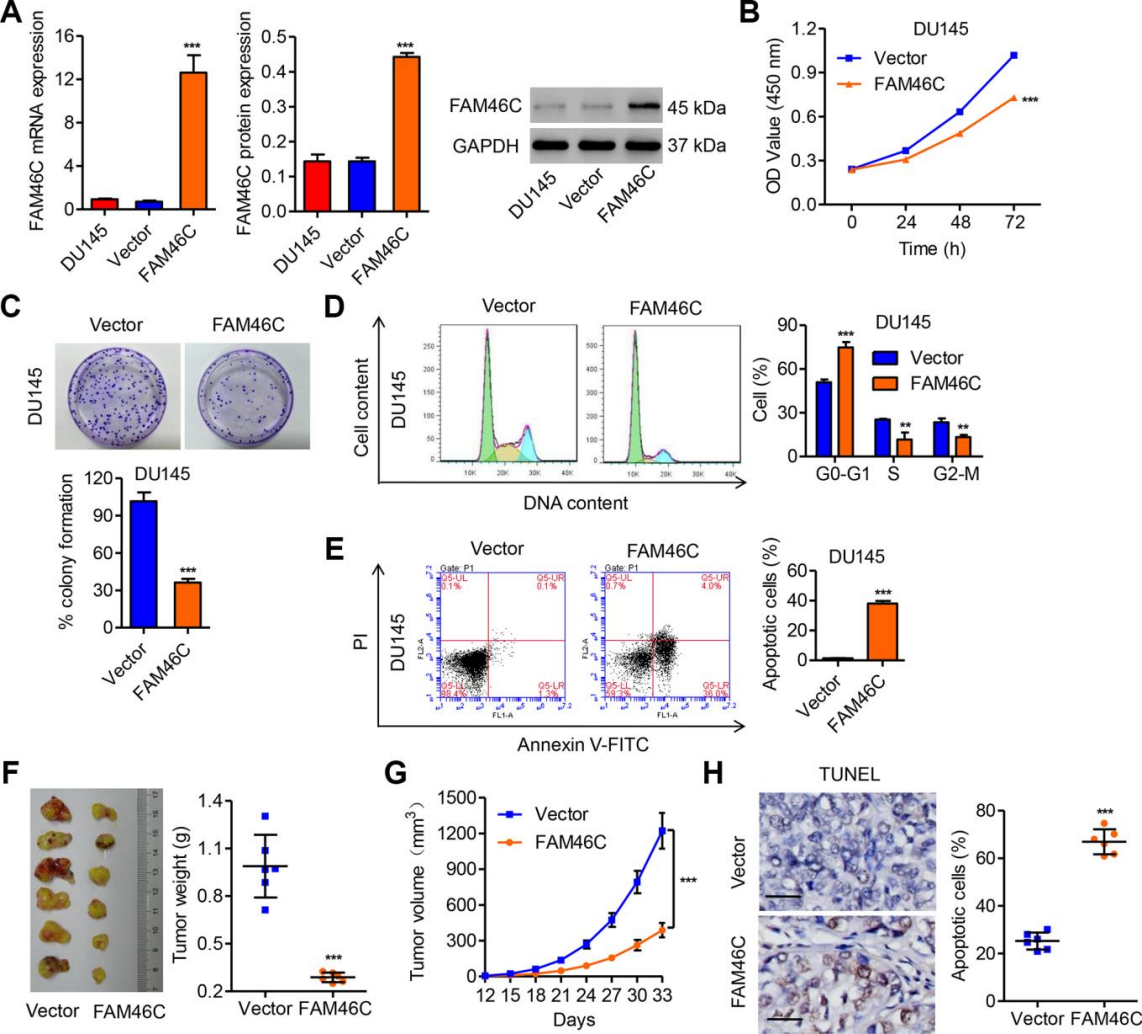
**FAM46C overexpression inhibited cell growth of DU145 and tumor growth *in vivo*.** (**A**) The efficiency of pLVX-Puro-FAM46C in overexpression of FAM46C in DU145 cells was measured by qPCR and western blot. After DU145 cells were transduced with pLVX-Puro-FAM46C, the cell proliferation (**B**, **C**), cell cycle (**D**) and apoptosis (**E**) were measured by CCK-8, colony formation and flow cytometry, respectively. After DU145 cells transduced with pLVX-Puro-FAM46C were injected into the nude mice (n=6), the tumor weight (**F**) and volume (**G**) was evaluated, and the apoptosis (**H**) was measured by TUNEL. Scale bars: 20 μm. **P<0.01, ***P<0.001 compared with Vector.

After DU145 cells were transduced with pLVX-Puro-FAM46C (Figure 4A), the cell proliferation was significantly inhibited by 16.3%, 23.2% and 28.3% at 24, 48 and 72 h, respectively, compared with vector (Figure 4B). Colony-formation assay showed that FAM46C overexpression significantly reduced the colony forming growth of DU145 cells by 64.3% compared with vector (Figure 4C). Moreover, FAM46C overexpression in DU145 cells significantly decreased the cell number in S and G2/M phase and increased the cell number in G0-G1 phase compared with vector (Figure 4D). FAM46C overexpression also promoted DU145 cell apoptosis by 25.9-fold compared with vector (Figure 4E). To evaluate the function of FAM46C in prostate cancer *in vivo*, DU145 cells transduced with pLVX-Puro-FAM46C or blank pLVX-Puro were injected into the nude mice. We found that the mice with pLVX-Puro-FAM46C injection showed decreased tumor weight and tumor volume, but increased cell apoptosis (Figure 4F–4H). These data indicated that FAM46C inhibited prostate cancer cell growth, thus playing a critical role in prostate cancer development.

### FAM46C inhibited cell cycle and promoted apoptosis and the PTEN signaling pathway

**Figure 5:**
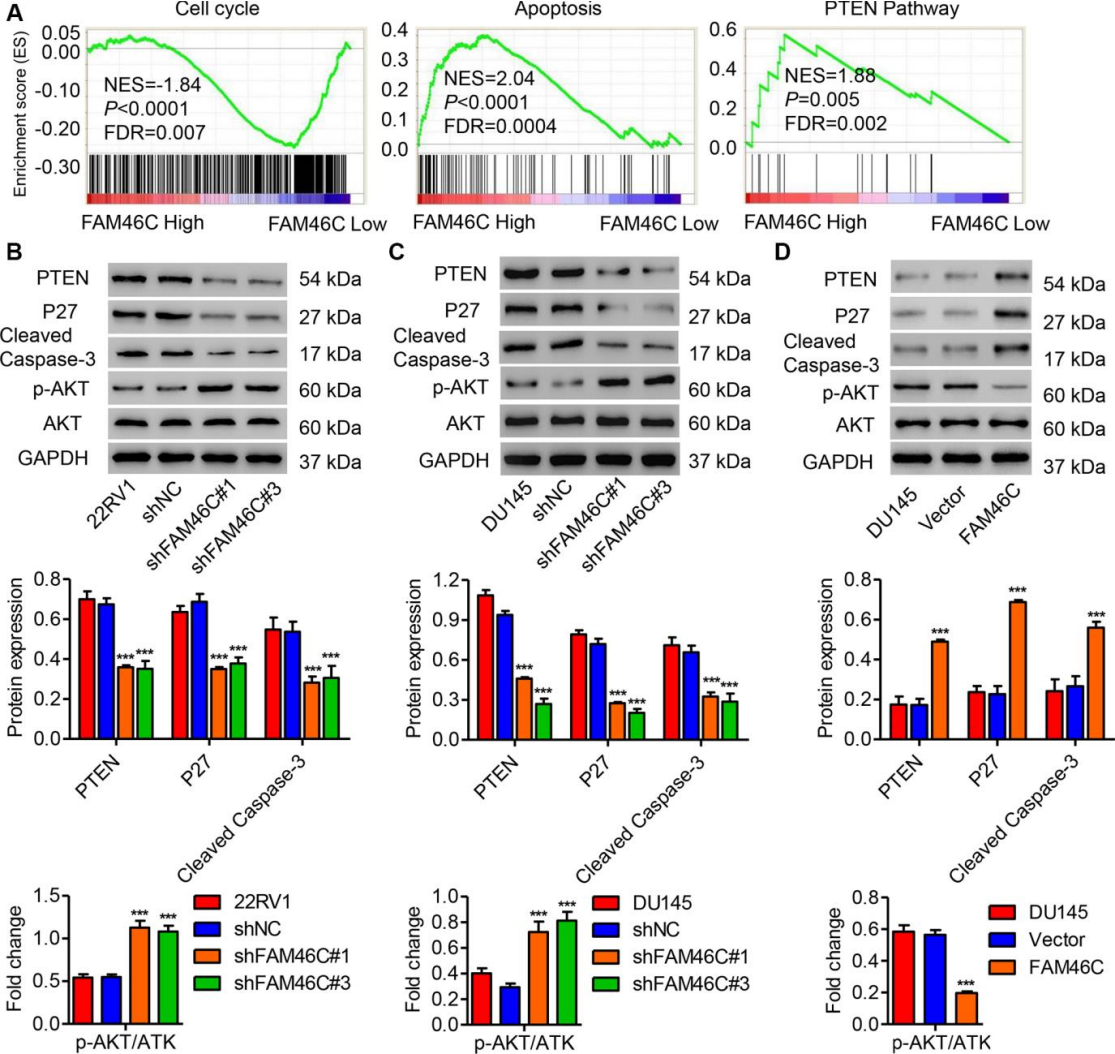
**FAM46C expression was associated with cell cycle, apoptosis and PTEN signaling pathways.** (**A**) GSEA analysis in patients with prostate cancer with higher versus lower FAM46C expression based on TCGA database showed that cell cycle, apoptosis and PTEN pathways were correlated with FAM46C expression. NES: normalized enrichment score. After 22RV1 (**B**) and DU145 cells (**C**) were transduced with pLKO.1-shFAM46C#1 and pLKO.1-shFAM46C#3, while DU145 cells (**D**) were transduced with pLVX-Puro-FAM46C, the expression of PTEN, P27, cleaved Caspase-3, AKT and p-AKT was measured by western blot. ***P<0.001 compared with pLKO.1-shNC or Vector.

Because GSEA data based on the TCGA database indicated that FAM46C expression is negatively correlated with cell cycle and positively correlated with cell apoptosis and PTEN signaling pathways in prostate cancer (Figure 5A), some proteins related to these pathways were measured by western blot. As shown in Figure 5B and 5C, FAM46C knockdown in 22RV1 and DU145 cells significantly inhibited the expression of PTEN, P27 and cleaved Caspase-3, but increased phosphorylation level of AKT. However, FAM46C overexpression in DU145 cells demonstrated an inverse effect (Figure 5D).

### The involvement of PTEN/AKT signaling pathway in the function of FAM46C in prostate cancer

**Figure 6:**
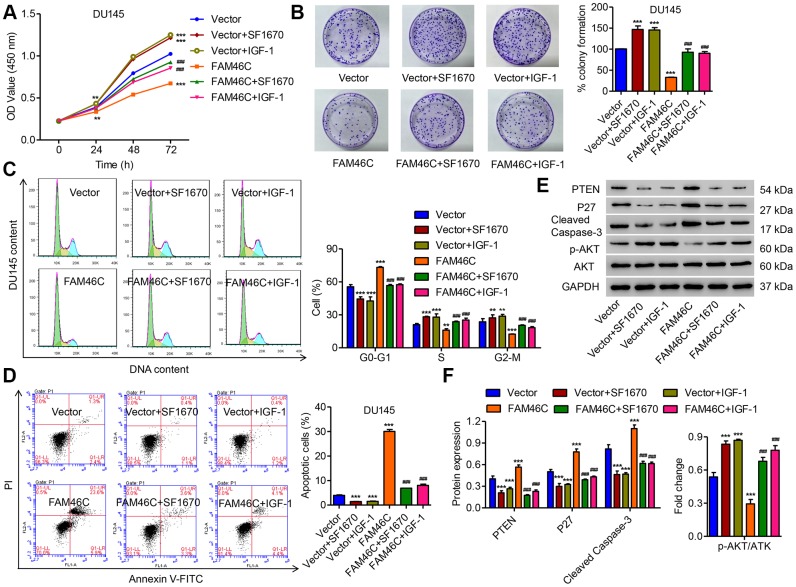
**FAM46C inhibited cell growth of DU145 cells via the PTEN/AKT signaling pathway.** After DU145 cells were transduced with pLVX-Puro-FAM46C with or without SF1670 or IGF-1, the cell proliferation (**A**, **B**), cell cycle (**C**), cell apoptosis (**D**) and expression of PTEN, P27, cleaved Caspase-3, AKT and p-AKT (**E**, **F**) were measured by CCK-8, colony formation, flow cytometry and western blot, respectively. **P<0.01, ***P<0.001 compares with vector; ^###^*P*<0.001 compared with FAM46C.

Based on the regulatory effect of FAM46C on the expression of PTEN and the level of p-AKT, we hypothesized that the PTEN/AKT signaling pathway may involve in the function of FAM46C in prostate cancer tumorigenesis. Consequently, the DU145 cells transduced with pLVX-Puro-FAM46C were treated with PTEN inhibitor SF1670 or AKT signaling agonist IGF-1, respectively. As shown in Figure 6A–6F, SF1670 or IGF-1 treatment significantly inhibited FAM46C overexpression-mediated cell growth and the expression of P27 and cleaved Caspase-3. These data indicate that FAM46C may inhibit prostate cancer cell growth through PTEN/AKT signaling pathway.

### FAM46C promoted PTEN expression through inhibiting PTEN ubiquitination

**Figure 7:**
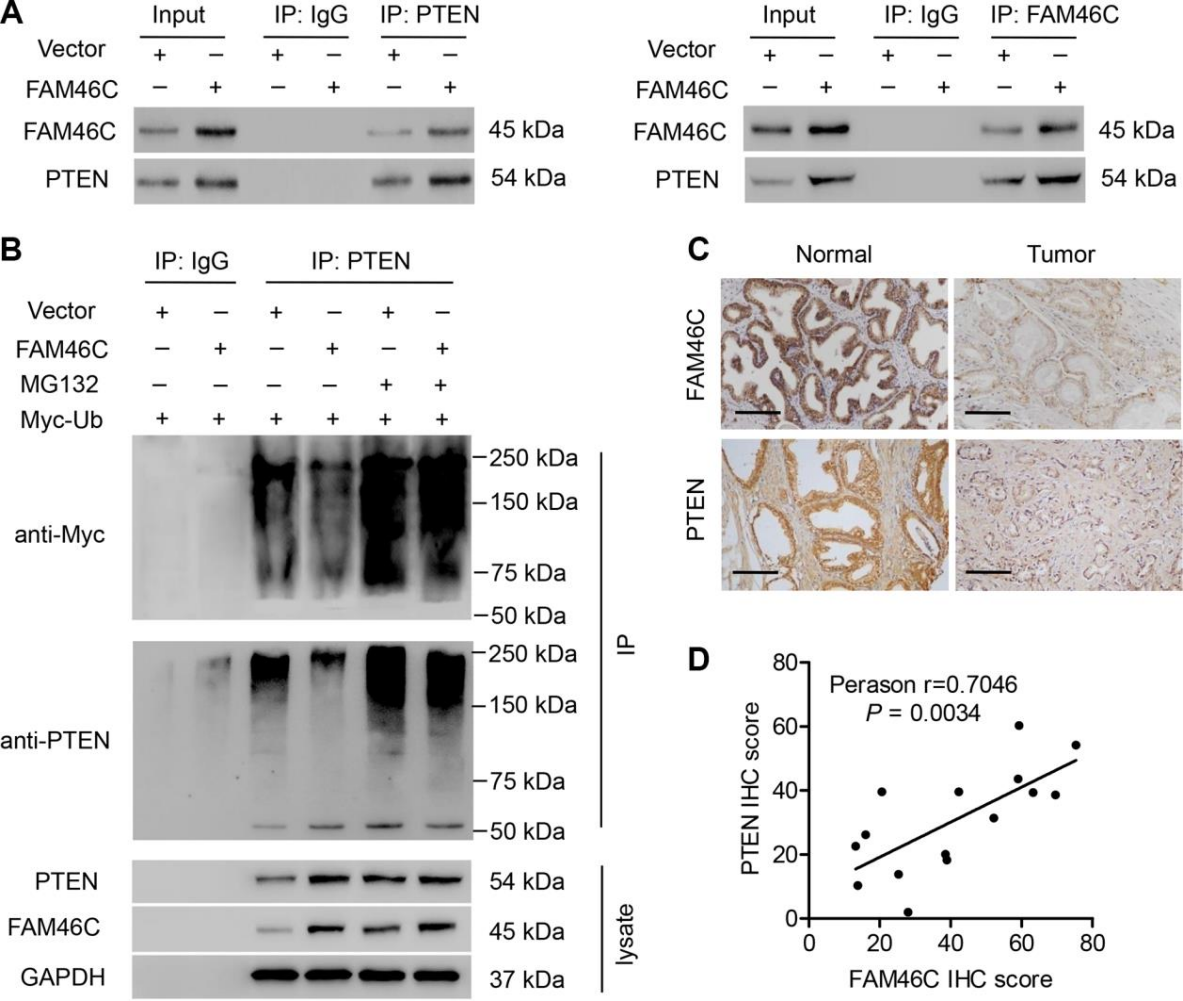
**The correlation between FAM46C and PTEN.** (**A**) Co-IP showed that FAM46C interacted with PTEN in DU145 cells. (**B**) PTEN was immunoprecipitated and immunoblotted in DU145 cells with 10 μM MG132 treatment and pLVX-Puro-FAM46C or blank pLVX-Puro transduction. (**C**) PTEN protein expression in prostate cancer tissues and noncancerous prostate tissues from hospital cohort were measured by immunohistochemistry. Scale bars: 100 μm. (**D**) Linear regression showed that FAM46C protein expression was positively correlated with that of PTEN (n=15).

Since FAM46C expression did not affect the mRNA expression of PTEN in prostate cancer cells (data not shown), we indicated that FAM46C may increase PTEN expression by the post-transcriptional modification. As shown in Figure 7A, Co-IP analysis demonstrated that FAM46C was interacted with PTEN in DU145 cells. Additionally, FAM46C overexpression in DU145 cells inhibited the ubiquitination of PTEN was significantly inversed by MG132 treatment, suggesting that FAM46C may inhibit the proteasome-dependent ubiquitination of PTEN (Figure 7B). To further investigate the correlation between FAM46C and PTEN, the expression of PTEN in prostate cancer was also measured. We found that the protein expression of PTEN was obviously decreased in prostate cancer tissues compared with noncancerous prostate tissues (Figure 7C). Linear regression showed that FAM46C protein expression was positively correlated with that of PTEN in prostate cancer tissues (Figure 7D).

### Higher FAM46C expression enhanced the chemosensitivity to docetaxel

**Figure 8:**
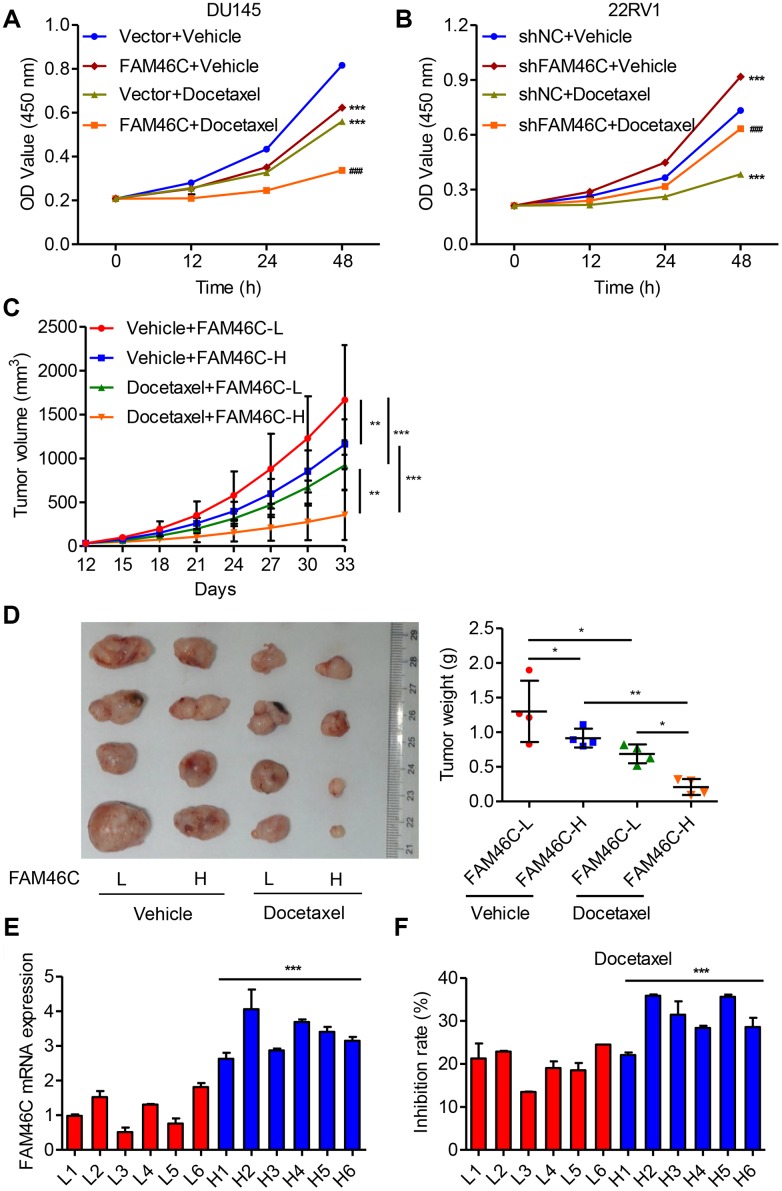
**Higher FAM46C expression enhanced the chemosensitivity of prostate cancer cells.** (**A**) CCK-8 showing the effect of pLVX-Puro-FAM46C lentivirus transduction on the proliferation of DU145cells treated with 10 nM docetaxel. (**B**) CCK-8 showing the effect of pLKO.1-shFAM46C transduction on the proliferation of 22RV1 cells treated with 10 nM docetaxel. ***P<0.001 compares with vector+vehicle; ^###^*P*<0.001 compared with FAM46C+vehicle. (**C**, **D**) Tumor weight and volume were measured in mice with PDX model following 10 mg/kg docetaxel chemotherapy (n=4). (**E**) FAM46C mRNA expression levels were measured by qPCR in the tumor cells isolated from primary prostate cancer patients from hospital cohort (n=12). (**F**) After primary isolated prostate cancer cells (n=12) were treated with 10 nM docetaxel, the inhibition rates of cell proliferation was measured (24 h relative to 0 h). *P<0.05, **P<0.01, ***P<0.001.

To examine whether FAM46C plays a role in prostate cancer response to chemotherapy, DU145 cells transduced with pLVX-Puro-FAM46C or blank pLVX-Puro lentivirus, while 22RV1 cells transduced with pLKO.1-FAM46C shRNA or pLKO.1-shNC, were treated with docetaxel. CCK-8 assay showed that DU145 cells with FAM46C overexpression were more sensitive to docetaxel (Figure 8A). Conversely, 22RV1 cells with FAM46C knockdown resulted in increased resistance to docetaxel (Figure 8B). PDX mice following docetaxel chemotherapy with high-FAM46C-expressing demonstrated smaller tumor size and tumor volume than that with low-FAM46C-expressing (Figure 8C and 8D). Moreover, the expression of FAM46C in the cells isolated from primary prostate cancer patients in hospital cohort was measured by qRT-PCR and categorized into FAM46C high and low groups (Figure 8E). In addition, the primary prostate cancer cells with high FAM46C expression were more sensitive to docetaxel than that with low FAM46C expression (Figure 8F). These data suggest that FAM46C may involve in chemosensitivity of prostate cancer.

## DISCUSSION

Prostate cancer is the most common non-cutaneous malignancy in the world, with many controversial aspects of treatment, and identifying genetic and molecular events that can help early detection of prostate cancer or possibly as a target for treatment are the top priority [27, 28]. In the present study, we investigated the biological function of FAM46C in prostate cancer. Clinical data showed that FAM46C was decreased and correlated with cell cycle, apoptosis and PTEN signaling pathway in prostate cancer. FAM46C overexpression inhibited prostate cancer cell growth and increased the chemosensitivity both *in vivo* and *in vitro*. Additionally, PTEN/AKT signaling pathway was involved in the function of FAM46C in prostate cancer tumorigenesis.

FAM46C belongs to a family of FAM46 metazoan-specific proteins, which has 4 members in human including FAM46A, FAM46B, FAM46C and FAM46D that are very similar in protein level, with sequence homology of at least 56.9% [9], indicating their similar molecular functions, such as RNA binding, polynucleotide adenylyltransferase activity, protein binding, transferase activity, and nucleotidyltransferase activity, enhancing mRNA stability and gene expression, and especially targeting mRNAs encoding endoplasmic reticulum-targeted protein and may be involved in induction of cell death. FAM46C as a tumor suppressor whose expression was decreased in multiple myeloma [9], gastric cancer [10], myeloma [11] and HCC [12], which was in line with our findings both in prostate cancer tissues and cell lines. Previous study reported that FAM46C was identified with potential pathogenic and prognostic significance based on the occurrence of recurrent homozygous deletions and mutations in myeloma [11]. Similarly, our results strongly suggested that FAM46C expression could be of clinical value as a prognostic indicator of several cancer types, such as BLCA, CSCC, HNSC, KIRC, LUAD, OC, PDAD, SARC or UCEC, and may as an independent prognostic factor play important role in prostate cancer. The present results in view of the loss- and the gain-function suggest the anti-proliferative and pro-apoptotic role of FAM46C in prostate cancer cells, which was consistent with our GSEA bioinformatics prediction and the previous studies [9, 29]. However, FAM46C overexpression in HCC cells induced the cell cycle G2-M phase arrest, but not the G0-G1 phase [12].

Most proteins involved in signal transduction system are products of oncogenes or tumor suppressors. Their abnormal expression is closely related to some diseases, especially the occurrence and development of cancers. PTEN is associated with the negative regulation of multiple signaling pathways, affecting target molecules and downstream signaling cascades, and regulating various physiological activities [30, 31]. Previous study found that the occurrence of prostate cancer and the process of hormone-independent transformation are related to the deletion of PTEN expression [32]. The deletion of PTEN expression occurs during the development of 20%-40% of primary prostate cancer and PTEN expression was observed in 30% of the hormone-independent prostate cancer tissues [33, 34]. Similarly, our results also observed decreased PTEN expression in prostate cancer tissues. Interestingly, the protein expression of FAM46C was found positively correlated with that of PTEN, and it promoted PTEN expression through inhibiting ubiquitination of PTEN. PTEN inhibiting AKT signaling has been observed in prostate cancer and is associated with cell cycle procession, cell apoptosis and cell proliferation [25, 26]. In the present study, our data suggested that PTEN/AKT signaling was involved in the function of FAM46C in prostate cancer. In line with our findings, previous studies showed that deletion of PTEN decreased P27 expression and Caspase-3 activation and promoted cell proliferation through activating PI3K/AKT pathway [35–37].

Furthermore, the biological function of FAM46C in hypersensitizing prostate cancer cell lines and PDX model mice to docetaxel is potentially important. Because prostate cancer chemotherapy is based primarily on the use of docetaxel, therapeutic increasing FAM46C expression can be combined with existing therapies to improve therapeutic efficacy. Increasing evidences have been shown that targeting PTEN and PI3K/AKT signaling pathway can inhibit or enhance chemosensitivity of several cancer types, including prostate cancer [24, 38, 39]. Therefore, we suggest that FAM46C may increase the chemosensitivity of prostate cancer cells to docetaxel through the PTEN/AKT signaling pathway, even though their roles are required for further investigation. FAM46C was found significantly increased in response to norcantharidin which can inhibit cell growth by inducing cell apoptosis and show anti-metastatic effect on several cancer cells [13]. Likewise, FAM46C was found to inhibit HCC cell migration and invasion. To the best of our knowledge, the role played by metastasis in prostate cancer has been proved [40]. Therefore, we may speculate that FAM46C highlighted its anti-metastatic role in prostate cancer. In the future investigation, we may focus on the FAM46C and metastasis in prostate cancer progression after chemotherapy.

In summary, our data indicate that FAM46C was associated with prognosis of prostate cancer patients and its overexpression suppressed cell growth of prostate cancer through PTEN/AKT signaling pathway, which may serves as a mode that mediates prostate cancer cell response to chemotherapy. FAM46C allowed an increased PTEN expression by inhibiting ubiquitination of PTEN. However, the mechanisms identified here deserve attention due to lack of specific mechanism between FAM46C and PTEN/AKT signaling pathway. This study suggests that FAM46C can be a possible therapeutic agent, particularly in combination with anti-cancer chemotherapy agents.

## MATERIALS AND METHODS

### Clinical samples

This study recruited 283 pathologically diagnosed prostate cancer patients and 15 patients with benign prostatic hyperplasia who received surgical resection from 2010-2014 in the Affiliated Hangzhou First People's Hospital, Zhejiang University School of Medicine (Hangzhou, China). All of the patients provided signed informed consent. The medical ethics committee of Affiliated Hangzhou First People's Hospital, Zhejiang University School of Medicine (Hangzhou, China) approved the present retrieval method of cancer specimens.

### Bioinformatics

RNA-sequencing dataset of prostate cancer cohort was downloaded from The Cancer Genome Atlas (TCGA, https://tcga-data.nci.nih.gov/tcga/) and analyzed by gene set enrichment analysis (GSEA) software version 2.0 as previously described [41]. The effect of FAM46C on survival in several cancer types was analyzed in the Kaplan Meier-plotter (http://www.kmplot.com/).

### Immunohistochemistry

The prostate cancer tissues samples (1.5 cm × 1.5 cm x 0.3 cm) were fixed in 10% formalin for 10 min, dehydrated in a gradient of ethanol for 2 h, and then transparent, paraffin and embedded. The slides (4-7 μm) were deparaffinized, rehydration and antigen-retrieved, after which slides were blocked by 3% H_2_O_2_ for 10 min and incubated with anti-FAM46C (Abcam, Cambridge, MA, USA) or anti-PTEN antibody (Abcam) at 25°C for 1 h and then stained with horseradish peroxidase (HRP)-labeled IgG (Shanghai Long Island Biotec. Co., Ltd, China) at 25°C for 20-30 min. Subsequently, the sections were stained with diaminobenzidine (DAB), counterstained with hematoxylin for 3 min and washed in water for 10 min. The tumor cells with positive staining more than 25% were defined as FAM46C or PTEN high expression group and that less than 25% were defined as FAM46C or PTEN low expression group.

### Cell culture

Human prostate cancer cell lines 22RV1, PC-3, LNCaP, and DU145 obtained from Cell Bank of Chinese Academy of Science (Shanghai, China) were cultured with RPMI-1640 medium (HyClone Laboratories, Inc., Logan, UT, USA). Human prostate epithelial cell line RWPE-2 obtained from American Type Culture Collection (ATCC) was cultured with DMEM. The cells cultured in the medium containing 10% fetal bovine serum (Gibco, Carlsbad, CA, USA) and 1% antibiotic (mixtures of penicillin and streptomycin, Solarbio, Shanghai, China) were maintained in a humidified incubator at 37°C and 5% CO_2_.

### Cell transfection

FAM46C (NM_017709.3) interference sequences (position 222-240: 5’-CCAGGGATTGCATGTCCTT-3’; position 308-326: 5’-GGACGAGGCAACTTTCCAA-3’ position; 1296-1314: 5’-GCAACTTCAGCAACTACTA-3’) and a scramble control shRNA (shNC, 5’-GCGTCACTCAACATACATA-3’) were synthesized by Sangon Biotech Co., Ltd. (Shanghai, China) and cloned into pLKO.1 vector to knockdown FAM46C expression. FAM46C overexpression plasmids were constructed by cloning full-length human FAM46C into the lentiviral expression vector pLVX-Puro (Addgen, Cambridge, MA, USA). Recombinant vectors (1000 ng) along with the psPAX2 (100 ng) and pMD2G (900 ng) packaging plasmids were co-transfected into 293T cells using Lipofectamine 2000. At 48 h after transfection, the supernatant was collected and transduced into 22RV1 or DU145 cells in the absence or presence of PTEN inhibitor SF1670 (250 nM; Selleck, Houston, TX, USA) or AKT signaling agonist IGF-1 (100 ng/ml; Peprotech, Rocky Hill, NJ, USA). Cells with pLKO.1-shNC or blank pLVX-Puro transduction were used as negative control.

### Cell counting kit-8 (CCK-8) assay

CCK-8 assay was performed using a Cell Proliferation and Cytotoxicity Assay Kit (SAB, CP002). Briefly, DU145 and 22RV1 cells (1×10^3^ cells per well) were seeded in the 96-well plates. After treatment, cells were incubated with 10 μL of CCK-8 solution. Cell proliferation was evaluated using the absorbance at 450 nm.

### Colony formation assay

After treatment, DU145 and 22RV1 cells were seeded in 10 cm dishes and cultured for two weeks. At the end of the incubation, colonies were fixed with paraformaldehyde for 15 min and stained with 0.5% crystal violet for 30 min. Colonies with 50 cells or more were counted.

### Flow cytometry analysis

DU145 and 22RV1 cells with the density of 3×10^5^ cell/well were seeded in 6-well plate and maintained at 37°C for one day. For cell cycle assay, cells were centrifugation at 1000×g for 5 min, fixed with 700 μL pre-cooled absolute ethyl alcohol, incubated with 1 mg/mL of RNase A (100 μL) in dark for 30 min and stained with 50 μg/mL of propidium iodide (PI, 400 μL) for 10 min. For cell apoptosis assay, cells were centrifugation at 1000×g for 5 min and incubated with 5 μL Annexin V-FITC for 15 min and 5 μL PI for 5 min at 4°C. The cell cycle and apoptosis were assayed on flow cytometer (Becton-Dickinson FACS Calibur, San Joes, CA, USA).

### Quantitative real-time PCR (qPCR)

Extraction of total RNA from prostate cancer tissues or cell lines was achieved by using the RNeasy Plus Mini Kit (Qiagen, Hilden, Germany) and reversely transcribed using TaqMan reverse transcription kit (Applied Biosystems, Palo Alto, CA, USA). Real-time PCR was performed using the SYBR Green qRT-PCR kit (Promega, Madison, WI, USA) on an ABI 7500 system following the manufacturer’s instructions. The primers used in the present study were subsequently shown: FAM46A-F, 5’-CCTGAGCGAGACCATTCCG-3’ and FAM46A-R, 5’-GAGGTCCAGGTCCTTGTAGCC-3’; FAM46B-F, 5’-AACAAGAGCGGCAAGAACG-3’ and FAM46B-R, 5’-CAGACATGGGAGTGGACGAG-3’; FAM46C-F, 5’-CGCCGTATAAGAACGGAG-3’ and FAM46C-R, 5’-TAGAAGAGGAGGCGACAG-3’; FAM46D-F, 5’-ATCTCCCTTTCAAATAACAC-3’ and FAM46D-R, 5’-GCCACCACCTCTAATCTC-3’; PTEN-F, 5’-TCAGGCGAGGGAGATGAGAG-3’ and PTEN-R, 5’-CAGGAGAAGCCGAGGAAGAG-3’; GAPDH-F, 5’-AATCCCATCACCATCTTC-3’ and GAPDH-R, 5’-AGGCTGTTGTCATACTTC-3’. Quantification of relative expression was normalized using GAPDH expression values and the 2^-ΔΔ Ct^ method was used to calculate relative gene expression.

### Immunoblotting analysis

Total protein was collected by using RIPA lysis buffer for 30 min at 4°C containing protease inhibitors, and the homogenates were centrifuged at 12,000×g for 20 min at 4°C. 15 μl of proteins were separated by 10-12% SDS-PAGE and transferred into nitrocellulose membrane (Millipore, Billerica, MA, USA). After blocking with 5% fat-free milk overnight at 4°C, the blots were incubated with anti-FAM46C (1:1000; Cat No. ab222808; Abcam), anti-PTEN (1:1000; Cat No. #9552; Cell Signaling Technology, Danvers, MA, USA), anti-p27 (1:1000; Cat No. #2552; Cell Signaling Technology), anti-cleaved Caspase-3 (1:500; Cat No. ab2302; Abcam), anti-p-AKT (1:1000; Cat No. #9271; Cell Signaling Technology), anti-AKT (1:1000; Cat No. #9272; Cell Signaling Technology), and anti-GAPDH (1:2000; Cat No. #5174; Cell Signaling Technology) antibody overnight at 4°C. The blots were then incubated with secondary antibodies conjugated with HRP (1:1000; Cat No. A0208, A0181 and A0216; Beyotime Biotechnology, Shanghai, China) for 1 h at 37°C. Protein expression was assessed by an enhanced chemiluminescence (ECL) kit (ECL New England Biolabs, Ltd, Whitby, ON, USA) following the manufacturer’s instructions.

### Co-immunoprecipitation (Co-IP) assay

Co-IP was performed as previously described [42]. Briefly, cold PBS was used to wash the cells for three times, and the cells were scraped into lysis buffer containing complete protease inhibitors and centrifuged at 14,000×g for 20 min at 4°C. The supernatants were incubated with anti-PTEN or normal IgG antibody, and the immunocomplexes were then associated with protein A-sepharose. After washing, the immunocomplexes were separated by SDS-PAGE. Immunoblotting was performed following standard procedures.

### *In vivo* deubiquitination assay

Cells transfected with the FAM46C expression vector were treated with or without MG132 for 4 h before harvest. *In vivo* deubiquitination assay was performed as previously described [43]. Briefly, cells were scraped into lysis buffer and centrifuged to remove cell debris. The cell extracts were subjected to immunoprecipitation with the indicated antibodies for 4 h at 4°C. After washing, the immunocomplexes were separated by SDS-PAGE and blotted with indicated antibodies.

### *In vivo* tumor growth

For *in vivo* tumorigenesis assay, a total of 4×10^6^ DU145 cells stably transduced with pLVX-Puro-FAM46C or blank pLVX-Puro were trypsinized, resuspended in PBS, and then subcutaneously injected into the flanks of BALB/c male nude mice (4-5 week-old; 6 per group; Shanghai Experimental Animal Center, Shanghai, China). Animals were sacrificed at 33 days after the injection, and the cell apoptosis was monitored by TUNEL staining as previously described [44]. To establishment of patient-derived xenograft (PDX) model, tumor tissues from prostate cancer patients, which divided into two groups according to the FAM46C expression (high vs low according to IHC staining), were collected at the time of surgery at Affiliated Hangzhou First People's Hospital, Zhejiang University School of Medicine (Hangzhou, China). These tissues (about 5×5×3 mm^3^) were subcutaneously transplanted into 6-8-week-old nude mice within 1 h of removal of tissues. Docetaxel (10 mg/kg; every 3 days) chemotherapy was initiated when tumor volumes reached around 700 mm^3^ (n=4 per group). At 33 days after injection, mice were sacrificed and tumor size was monitored. Animal experiments were approved by the Affiliated Hangzhou First People's Hospital, Zhejiang University School of Medicine (Hangzhou, China) institutional ethical committee and performed according to the legal requirements.

### Statistical analysis

Data are presented as mean ± SD, and each test was repeated at least three times. Statistical analysis was conducted using Student’s t-test or one-way or two-way ANOVA with GraphPad Prism software, version 7.0 (GraphPad Software, USA). *P* < 0.05 was regarded as statistically significant.
